# Usefulness of 3D CT/MRI Fusion Imaging for the Evaluation of Lumbar Disc Herniation and Kambin’s Triangle

**DOI:** 10.3390/diagnostics12040956

**Published:** 2022-04-12

**Authors:** Masakazu Nagamatsu, Praful Maste, Masato Tanaka, Yoshihiro Fujiwara, Shinya Arataki, Taro Yamauchi, Yoshiyuki Takeshita, Rika Takamoto, Tsukasa Torigoe, Masato Tanaka, Ryosuke Tanaka, Shinsuke Moriue

**Affiliations:** 1 Department of Radiology, Okayama Rosai Hospital, 1-10-25 Chikkomidorimachi, Minami Ward Okayama, Okayama 702-8055, Japan; n.masa1987215@gmail.com (M.N.); takerad@gmail.com (Y.T.); naskal.rika.15@gmail.com (R.T.); vv.mchukam@gmail.com (T.T.); masamaru.tanaka@gmail.com (M.T.); rsm19811102@gmail.com (S.M.); 2 Department of Orthopaedic Surgery, Okayama Rosai Hospital, 1-10-25 Chikkomidorimachi, Minami Ward Okayama, Okayama 702-8055, Japan; drprafulmaste@gmail.com (P.M.); fujiwarayoshihiro2004@yahoo.co.jp (Y.F.); araoyc@gmail.com (S.A.); ygitaro0307@yahoo.co.jp (T.Y.); 3 Department of Radiology, Hamamatsu University Hospital, 1-20-1 Handayama, Higashi Ward, Hamamatsu 431-3125, Japan; rtanaka@hama-med.ac.jp

**Keywords:** CT/MRI fusion image, endoscopic lumbar surgery, working space, lumbar nerve root, lumbar disc herniation, Kambin’s triangle

## Abstract

Study design: Prospective study. Objective: The aim of this study is to visualize the morphology of a lumbar herniated disc and Kambin’s triangle in three dimensions (3D) based on preoperative CT/MRI fusion images. Methods: CT/MRI fusion images of 23 patients (10 males and 13 females; mean age 58.2 years) were used to evaluate Kambin’s triangle, which is created between the superior articular process (SAP), exiting nerve root (ENR), inferiorly by the superior endplate of the lower lumbar vertebra and dural canal medially at 60 degree and 45 degree endoscopic approach angles. The percentage of the safe usage of transforaminal endoscopic approach was evaluated to utilize a 5 mm dilater without partial facet resection in the fusion image. The 3D lumbar nerve root sleeve angulation (3DNRA), which is the angle between the axis of the thecal sac and the nerve root sleeve, was calculated. The herniated discs were also visualized in the CT/MRI fusion image. Results: The 3DNRA became smaller from L2 to S1. The L2 3DNRA was statistically larger than those of the other root, and the S1 3DNRA was significantly smaller than the others (*p* < 0.05). (L2, 41.0°; L3, 35.6°; L4, 36.4°; L5, 33.9°; and S1, 23.2°). The SAP-ENR distance at 60° was greatest at L4/5 (5.9 mm). Possible needle passages at 60° to each disc level were 89.1% at L2/3, 87.0% at L3/4 and 84.8% at L4/5. However, the safe 5 mm dilater passage at 60° without bony resection to each disc level were 8.7% at L2/3, 28.3% at L3/4 and 37.0% at L4/5. The 60° corridor at L2/3 was the narrowest (*p* < 0.01). All herniated discs were visualized in the fusion image and the root compression site was clearly demonstrated especially with foraminal/extraforaminal herniations. Conclusion: The 3D lumbar CT/MRI fusion image enabled a combined nerve-bony assessment of Kambin’s triangle and herniated disc. A fully endoscopic 5 mm dilater may retract the exiting nerve root in more than 60% of total cases. This new imaging technique could prove to be very useful for the safety of endoscopic lumbar disc surgery.

## 1. Introduction

Fully endoscopic surgery and percutaneous transforaminal lumbar interbody fusion (PETLIF) have been accepted as a minimally invasive lumbar surgery (MIS) [1,2]. These approaches are through Kambin’s triangle, which is an anatomical corridor made by the exiting nerve root (ENR), dural canal, superior endplate of the lower lumbar vertebra and SAP [3]. For these techniques, one of the most common complications is an exiting nerve root injury [4]. To avoid this severe complication, anatomical considerations before surgery are very important [5]. 

There are several reports that discuss contraindications regarding access to Kambin’s triangle; however, these are cadaveric studies [5,6] or only preoperative MRI study [7]. Various cadaveric studies have reported approximately 80% of total Kambin’s triangles are impossible to access [6]. However, preoperative evaluation of Kambin’s triangle in patients is more important for endoscopic lumbar surgeons. Furthermore bony structures cannot be evaluated precisely by MRI.

CT/MRI fusion images have recently become available to evaluate spinal anatomy for decompression surgery [8] as well as the accessibility of the L5-S1 oblique approach to perform interbody fusion [9]. With this new CT/MRI fusion images, not only soft tissues such as the nerve root, vascular and intervertebral disc, but also bony structures can be evaluated simultaneously. We herein report the accessibility of Kambin’s triangle using a novel CT/MRI fusion technique that extracts nerve tissue and intervertebral disc images from magnetic resonance imaging (MRI) and bony images from 3D CT and fuses them into a composite image depicting all the structures in relation to one another.

## 2. Materials and Methods

### 2.1. Patient Population

**Figure 1:**
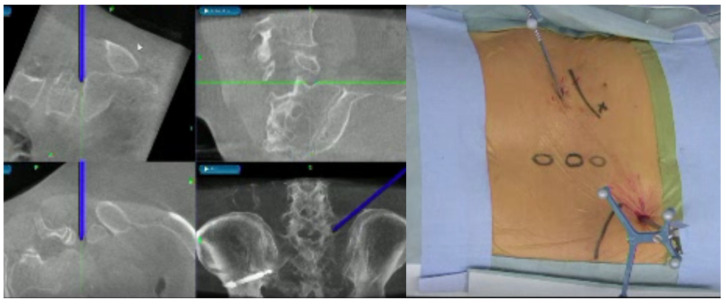
A 57-year-old female. Right L5-S1 lumbar disc herniation. Fully endoscopic discectomy.

The present study was approved by Okayama Rosai Hospital institutional review board (No. 332). We obtained fully informed consent from each patient prior to participation in this study. From June 2021 to January 2022, a total of 23 patients with lumbar disc herniation who underwent spinal surgery at our hospital were included in the present study (Table 1, Figure 1). The inclusion criteria for this study were: age ≥ 18 years and planned spinal surgery. We excluded spondylolisthesis, lumbar scoliosis > 5 degrees and history of previous spinal surgery.

### 2.2. Image Technique

Nerve and disc images were taken using Signa HDxt 1.5-T platform (General Electric, Boston, MA, USA). The image sequence was fast imaging employing steady-state acquisition (3D-FIESTA) from steady-state free precession (SSFP), using the following settings: slice width, 1.0 mm; slice interval, 0 mm; echo time, 2.1 ms; flip angle, 30°; matrix, 224 × 244; accumulations, 1; bandwidth, 62.50 Hz/pixel; field of view and 224 mm. The imaging range was from the upper end of the L1 vertebra to the lower end of the S2 vertebra. The number of slices was about 84, and the scan time was about 2.44 min.

The bone 3D image was created using the Aquilion lightning platform (Canon, Tokyo, Japan). Imaging conditions were: tube voltage, 120 kV; scan speed, 0.50 s; slice width, 0.5 × 80 mm; and helical pitch and 65.0. From these data, the 3D spine image was reconstructed using AIDR 3D enhanced eStrong software (Canon).

### 2.3. CT-MRI Fusion Image

**Figure 2:**
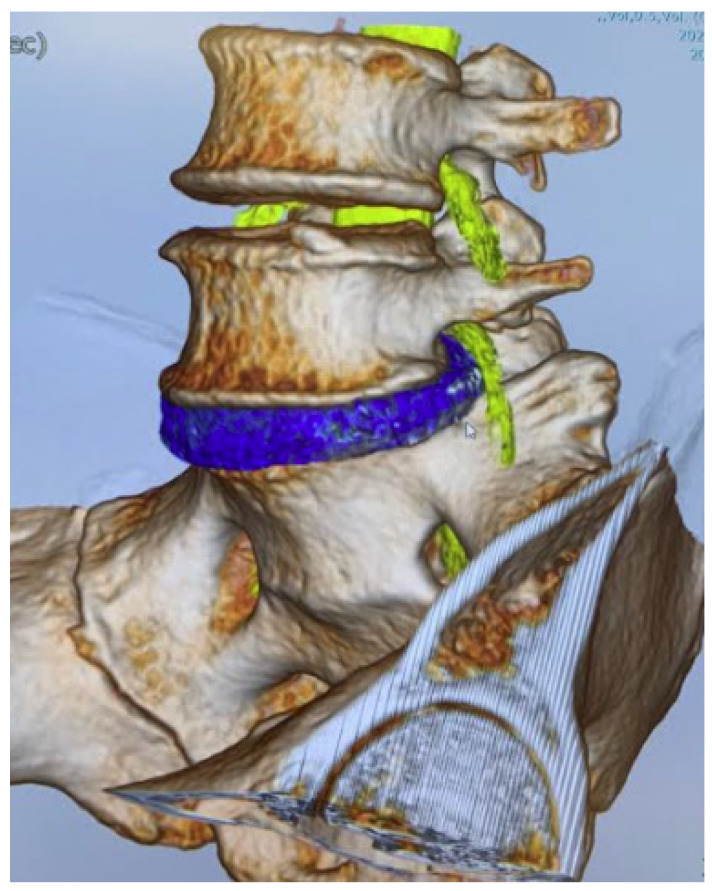
MRI and CT medical image fusion.

The fusion image combining 3D disc and nerve tissue image with the bony structure was obtained using Synapse Vincent version 6.4 software (Fujifilm, Tokyo, Japan). The first step was justification of the CT image (soft tissue mode) and MR image (FIESTA) in a 2D image using high signals from CSF and disc. The second step was obtaining the 3D bony image from CT, setting the transparency rate of the ilium bilaterally at 0.2 to make the image of the L1 to S2 level. With MRI, images of the neural tissue and disc were made manually. The final step was merging of the bony image from CT and the soft tissue image from MRI. The total time to create the CT/MRI fusion was approximately 5 min (Figure 2).

### 2.4. Evaluation of Merged Images

**Figure 3:**
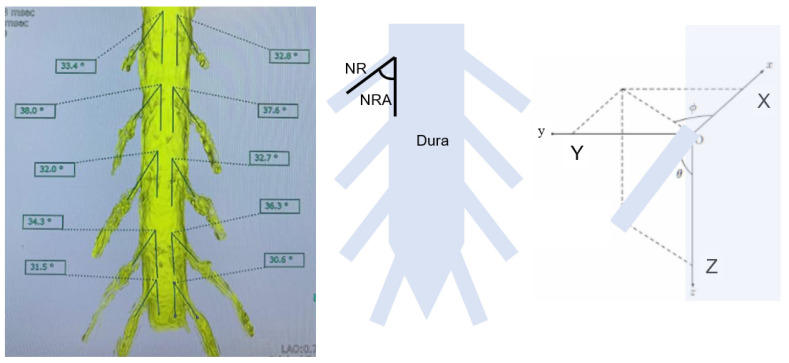
3-D nerve root angle (3DNRA).

**Figure 4:**
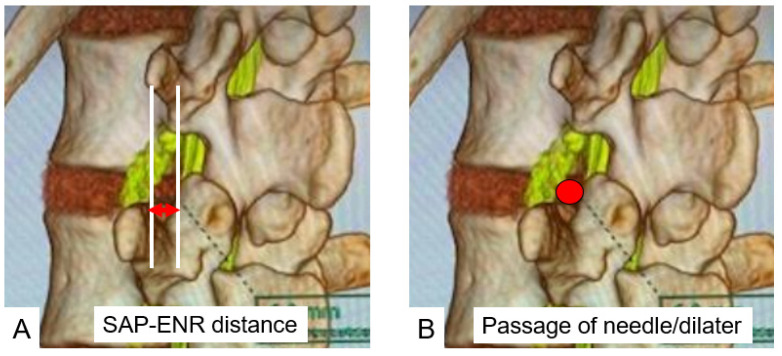
(**A**): SAP-ENR distance. (**B**): Passage of needle and dilater. A red arrow indicates SAP-ENR distance and a red dot shows passage of needle/dilater.

To reduce subjective errors in calculations, each image was justified, and measurements were made by two individuals. We measured the following values; (a) 3-D nerve root angle (3DNRA) from L2 to S1 (Figure 3); (b) the distance between the superior articular process and exiting nerve root at the lower endplate level (SAP-ENR distance), which is the base of Kambin’s triangle at 45 degrees and 60 degrees (Figure 4A); and (c) the accessibility of Kambin’s triangle with a needle (1 mm) and a small dilater (5 mm) (Figure 4B).

### 2.5. Statistical Analysis

All data are expressed as the mean ± standard deviation (SD). Imaging findings were statistically compared between the groups. The Mann–Whitney U test was used to analyze continuous variables, and the chi-squared test was used to analyze dichotomous variables. A *p* value < 0.05 was defined as statistically significant.

## 3. Results

### 3.1. 3-D Nerve Root Angle (3DNRA)

**Figure 5:**
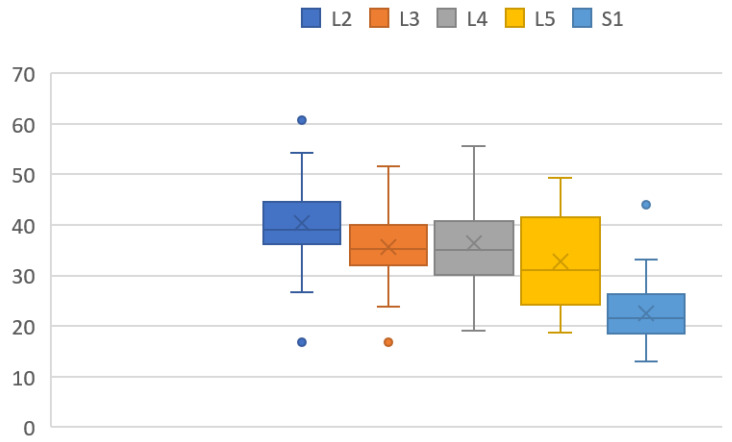
3-D nerve root angulation (3DNRA). The dots indicate outliers.

3DNRA became smaller from cranial to caudal (Figure 5). L2 3DNRA was statistically larger than those of the other root, and the S1 3DNRA was significantly smaller than the others (*p* < 0.05). (L2, 41.0°; L3, 35.6°; L4, 36.4°; L5, 33.9°; and S1, 23.2°).

### 3.2. Superior Articular Process to Exiting Nerve Distance (SAP-ENR Distance)

The mean distances between SAP and ENR at a 45° approach angle were 4.9 ± 2.5 mm at L2/3, 4.3 ± 2.3 mm at L3/4 and 6.8 ± 4.0 mm at L4/5. The mean distances between SAP and ENR at a 60° approach angle were 4.1 ± 2.1 mm at L2/3, 3.8 ± 2.4 mm at L3/4 and 5.9 ± 3.6 mm at L4/5. The SAP-ENR distances at 45° and 60° were the greatest at L4/5 (Table 2). 

### 3.3. Possible Needle (1 mm) Passage at 60° and 45°

Possible needle passages at 45° to each disc level were 96.7% at L2/3, 89.1% at L3/4, 94.5% at L4/5. However, the possible needle passages at 60° to each disc level were 89.1% at L2/3, 87.0% at L3/4 and 84.8% at L4/5 (Table 3).

### 3.4. Possible Dilater (5 mm) Passage at 60° and 45°

Possible dilater (5 mm) passages at 45° to each disc level were 50.0% at L2/3, 19.5% at L3/4 and 45.7% at L4/5. Possible dilater (5 mm) passages at 60° to each disc level were 8.7% at L2/3, 28.3% at L3/4 and 37.0% at L4/5. At 45° L3/4 was the most difficult for the 5 mm dilater passage compared with other levels (*p* < 0.01). On the other hand, at 60° L2/3 was the narrowest compared with other levels (*p* < 0.05) (Table 3).

### 3.5. Herniated Disc Visualization

**Figure 6:**
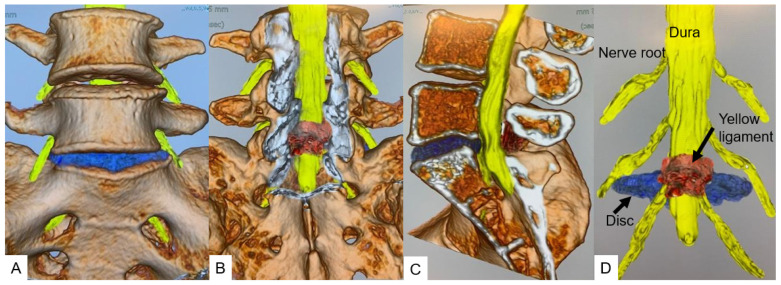
**A** 46-year-old male, left L5/S1 foraminal/extraforaminal disc herniation. (**A**): Anterior view. (**B**): Posterior view. (**C**): Lateral view. (**D**): Without bony structure.

**Figure 7:**
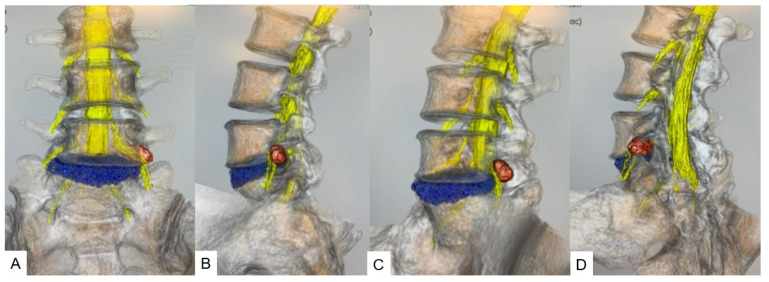
A 46-year-old male, left L5/S1 foraminal/extraforaminal disc herniation. (**A**): Anterior view. (**B**): Lateral view. (**C**): Anterior oblique view. (**D**): Posterior oblique view.

**Figure 8:**
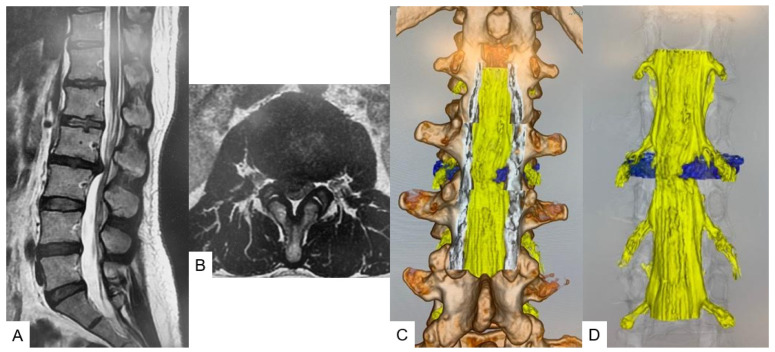
A 44-year-old female, right L2/3 paramedial disc herniation. (**A**): T2 weighted midsagittal MRI. (**B**): L2/3 T2 weighted axial MRI, (**C**): Posterior view. (**D**): Without bony structure.

All herniated discs were visualized in the fusion images, and the root compression site was clearly demonstrated in foraminal/extraforaminal disc herniations (Figure 6, Figure 7 and Figure 8).

## 4. Discussion

Surgery for lumbar disc herniation is the most common spinal surgery performed by spine surgeons. Since its first description in 1929 by Dandy [10], surgery for lumbar disc herniation has evolved tremendously due to various factors, i.e., the invention of the surgical microscope, better instruments and the development of diagnostic imaging, such as CT and MRI. In the last couple of decades, minimally invasive spine surgery has progressively become the mainstay of modern spine surgery for lumbar disc herniation as well as for more complicated spine problems, such as degenerative spine disease, spinal deformity, spinal tumors, etc. 

Fully endoscopic lumbar discectomy (FED) is one of the latest techniques in surgery for contained lumbar disc herniation [1,2]. The range of conditions that can be treated by endoscopic spine surgery has expanded, ranging from contained prolapsed intervertebral discs to noncontained migrated herniated discs, hard calcified discs, spinal stenosis in the central or lateral recess and the foraminal and extraforaminal region and other combinations of degenerative conditions requiring decompression or fusion surgery [11,12]. Endoscopic spine surgery provides safe, direct and targeted access to the compressive pathology with minimal soft tissue trauma while performing decompression and/or fusion [1,12]. 

Kambin’s triangle is an anatomical corridor in the lateral aspect of the lumbar spine bordered anteriorly by the exiting root, inferiorly by the proximal plate of the lower lumbar segment, posteriorly by the proximal articular process of the inferior vertebra and medially by the traversing nerve root and dural sac [13,14]. This triangle is also the site for transforaminal epidural steroid injection and diagnostic discograms along with being the corridor for endoscopic discectomy and spinal fusion surgeries [14,15]. 

Hijikata and Kambin separately introduced percutaneous nucleotomy, and Kambin further described the safe triangular zone for docking and working on the transforaminal region [12]. FED and percutaneous endoscopic transforaminal lumbar interbody fusion (PETLIF) are performed through this pathway to approach the intervertebral disc space in the lumbar spine [11]. This is an important landmark to avoid exiting root injury. The limited dimensions of Kambin’s triangle limit the size of the cannula and instruments that can be passed through it [7]. 

Evaluation of Kambin’s triangle is necessary as it is the most important factor that determines whether FED or any other endoscopic procedure is possible in a given patient or not [7,15]. Kambin’s triangle can be evaluated radiologically using CT and MRI scans. While CT will give the bony details, high resolution MRI will show the neurovascular outlay in and around Kambin’s triangle. Various authors have studied Kambin’s triangle and its various parameters in either cadavers or as pre-operative evaluation using CT or MRI [5,6,7].

In this study, the 3-D nerve root angle (3DNRA) became smaller moving from cranial to caudal. These results are similar to a previous MRI study [16]. Hasegawa et al. in 1993 performed a morphometric analysis of lumbosacral roots and dorsal root ganglia and their bony relations [16]. They studied the level of origin of the roots and the root takeoff angles (NRA). They reported progressively increasing NRA from L1 to L5 with NRA dipping slightly at S1. The dorsal root ganglion dimensions were the highest at S1. The pedicle width increased from L1 to L5, while the pedicle height showed a marginally decreasing trend in the cranio caudal direction. Our 3DNRAs at each level were slightly larger than their results because our measurement was 3D not 2D. 

Kambin’s triangles of 45° and 60° were the largest at the L4/5 disc level in our study. There have been several previous studies that evaluated Kambin’s triangle. Min et al., in a cadaver study, concluded that the mean diameter of the working zone base was 11.6 ± 4.6 mm, and this value increases going down the level of the spine. L2–L3, L3–L4 and L4–L5 intervertebral foramens were similar in multiple comparison tests [17]. Pairaiturkar et al. reported that the superior articular process to exiting nerve (SAP-ENR) distance at the vertebral endplate in 50 MRI-imaged patients was 7.15 ± 1.43 mm, 6.17 ± 1.96 mm and 7.62 ± 2.41 mm at L2/3, L3/4 and L4/5, respectively [7]. 

Zhang et al. measured the maximum SAP-ENR distance using cadavers and reported values of 5.13 ± 0.7 mm, 5.96 ± 0.4 mm and 6.47 ± 0.4 mm at L2/3, L3/4 and L4/5, respectively [18]. Ozer et al. classified Kambin’s triangle into three types closed, narrow and a normal triangle [6]. A wide and safe triangle may not exist in certain patients; hence, more care must be taken during FED to avoid nerve damage. However, these studies were cadaveric or 2-dimentional MRI studies. CT/MRI fusion image is very useful to evaluate Kambin’s triangle preoperatively because these images can evaluate soft tissues (nerve root and intervertebral disc) and bony structures simultaneously.

Yamada et al. evaluated Kambin’s triangle using 3D CT/MRI fusion images of the lumbar spine and nerves [11]. They were able to quantify the safety zone with respect to the position of the contents of the triangle as well as with respect to the size of the endoscopic cannula and the angle of approach. The SAP-ENR distance at 60° was the greatest at L4/5 and was significantly greater at L2/3 and L4/5 compared with at L3/4 (*p* < 0.01, *p* < 0.01, respectively). The SAP-ENR distance at 45° was the greatest at L2/3, and it was larger in L2/3 and L4/5 than in L3/4 (*p* < 0.01, *p* < 0.01, respectively). The SAP-ENR distances at L4/5 were significantly greater at 60° than at 45° (*p* < 0.01). 

One of the most important preoperative evaluation for Kambin’s triangle is the possibility of safe passage for endoscopic dilaters. In our results, a 5 mm dilater can be passed through Kambin’s triangle without any retraction only 37.0% at the L4/5 60° corridor. Thus, the endoscopic surgeons should always keep in mind that insertion of the dilater only also has a risk of exiting nerve traction or injury. Endoscopic foraminoplasty is the best option for a narrow Kambin’s triangle [19,20]. 3D CT and MRI fusion gives not only the anatomy but also a clear visual and measured view of Kambin’s triangle along with the position of the disc herniation in relation to the exiting and traversing roots and the feasibility of safely inserting a 5 mm dilator at 45 and 60 degrees through the triangle for PELD and PETLIF. 

This helps greatly in patient selection and hence the results. As is evident from this study, the various measured parameters in Kambin’s triangle can be asymmetrical on the left and right side. Hence, this method of evaluation helps in deciding whether fully endoscopic procedure can be done on both sides in a patient with bilateral nerve root compression. The thickness of the ligamentum flavum as it envelops the dural tube can be determined along with hypertrophy of the facet joints. 

Bony anomalies, such as lumbarization and sacralization, can be characterized in detail preoperatively due to the 360 degree assessment with our technique. We firmly believe that, over a period of time, this technique with 3D CT and MRI fusion will become an integral part of planning for fully endoscopic spinal surgery. It will be helpful in training younger spinal surgeons to understand the three dimensional spinal anatomy and help accelerate the learning curve.

There are several limitations of this study. The sample number was slightly small. It was somewhat difficult to create the same position to conduct the CT and MRI. As the spine bends with changes in body position, the anatomical dimensions may vary slightly when a patient is positioned prone. This can be circumvented to some extent by using a common patient positioning system during MRI, CT and during surgery. The muscle relaxation during general anesthesia can affect the spinal dimensions. The 3-D workstation (Vincent system) that created the CT/MRI fusion is costly, which will likely be a limiting factor in its availability and usage. 

## 5. Conclusions

The 3D lumbar CT/MRI fusion image enabled a combined soft tissue/bone assessment of Kambin’s triangle and herniated disc. A fully endoscopic 5 mm dilater was found to retract the exiting nerve root in more than 60% of total cases. This new technique could be useful not only for the safety of lumbar disc surgery but also for the analysis of human anatomy.

## Figures and Tables

**Table 1:** Patient demographics.

	N = 23
Gender (male:female)	10:13
Age (mean ± S.D.) (year)	58.2 ± 18.2
Height (mean ± S.D.) (cm)	160.6 ± 11.2
Body weight (mean ± S.D.) (kg)	63.5 ± 16.3
Body mass index (mean ± S.D.) (kg/m^2^)	24.4 ± 4.2

**Table 2:** Superior articular process for the exiting nerve distance.

Disc Level	Angle of Rotation (°)	Right (mm)	Left (mm)	Mean (mm)
L2/3	45	4.9 ± 2.3	5.7 ± 2.7	5.3 ± 2.5
L2/3	60	3.9 ± 2.0	4.3 ± 2.4	4.1 ± 2.1
L3/4	45	4.3 ± 2.3	4.3 ± 2.0	4.3 ± 2.3
L3/4	60	4.0 ± 2.8	3.7 ± 2.2	3.8 ± 2.4
L4/5	45	6.4 ± 3.9	7.1 ± 4.0	6.8 ± 4.0
L4/5	60	5.6 ± 3.8	6.1 ± 3.6	5.9 ± 3.6

**Table 3:** Possible needle (1 mm) and dilater (5 mm) passage.

Disc Level	Angle of Rotation (°)	Needle Passage (%)	Dilater Passage (%)
L2/3	45	96.7	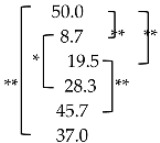
L2/3	60	89.1	
L3/4	45	89.1	
L3/4	60	87.0	
L4/5	45	94.5	
L4/5	60	84.8	

* *p* < 0.05 and ** *p* < 0.01.

## Data Availability

The data presented in this study are available in the article.
